# Two New 1,3,4-Oxadiazoles With Effective Antifungal Activity Against *Candida albicans*

**DOI:** 10.3389/fmicb.2019.02130

**Published:** 2019-09-12

**Authors:** Isis Regina Grenier Capoci, Karina Mayumi Sakita, Daniella Renata Faria, Franciele Abigail Vilugron Rodrigues-Vendramini, Glaucia Sayuri Arita, Admilton Gonçalves de Oliveira, Maria Sueli Felipe, Bernard Maigret, Patricia de Souza Bonfim-Mendonça, Erika Seki Kioshima, Terezinha Inez Estivalet Svidzinski

**Affiliations:** ^1^Department of Clinical Analysis and Biomedicine, The State University of Maringá, Maringá, Brazil; ^2^Laboratory of Electron Microscopy and Microanalysis, State University of Londrina, Londrina, Brazil; ^3^Department of Cellular Biology, The University of Brasília, Brasília, Brazil; ^4^LORIA, University of Lorraine, Nancy, France

**Keywords:** *Candida albicans*, thioredoxin reductase, *in vitro*, *in vivo*, antifungal activity

## Abstract

*Candida* infections have become a serious public health problem with high mortality rates, especially in immunocompromised patients, since *Candida albicans* is the major opportunistic pathogen responsible for systemic or invasive candidiasis. Commercially available antifungal agents are restricted and fungal resistance to such drugs has increased; therefore, the development of a more specific antifungal agent is necessary. Using assays for antifungal activity, here we report that two new compounds of 1,3,4-oxadiazoles class (LMM5 and LMM11), which were discovered by *in silico* methodologies as possible thioredoxin reductase inhibitors, were effective against *C. albicans*. Both compounds had *in vitro* antifungal activity with MIC 32 μg/ml. Cytotoxicity *in vitro* demonstrated that LMM5 and LMM11 were non-toxic in the cell lines evaluated. The kinetic of the time-kill curve suggested a fungistatic profile and showed an inhibitory effect of LMM5 and LMM11 in 12 h that remained for 24 and 36 h, which is better than fluconazole. In the murine systemic candidiasis model by *C. albicans*, the two compounds significantly reduced the renal and spleen fungal burden. According to the SEM and TEM images, we hypothesize that the mechanism of action of LMM5 and LMM11 is directly related to the inhibition of the enzyme thioredoxin reductase and internally affect the fungal cell. In view of all *in vitro* and *in vivo* results, LMM5 and LMM11 are effective therapeutic candidates for the development of new antifungal drugs addressing the treatment of human infections caused by *C. albicans*.

## Introduction

Although *Candida albicans* is present in the healthy population as commensal microbiota, this species is classified as an opportunistic fungus. Under favorable conditions, yeast can enter the bloodstream leading to deep-tissue infections (Dadar et al., 2018). Many types of candidiasis, such as systemic and invasive candidiasis, have increased in the last decades with high incidence in immunocompromised patients (Pfaller and Diekema, 2010; Colombo et al., 2014; Tedeschi et al., 2016), including significant morbidity and mortality in organ transplant patients (Bassetti et al., 2017; Diba et al., 2018). In the ICU, invasive candidiasis represents about 20% of all these infections (Lamoth et al., 2018). The general distribution of species is dependent on factors such as patient population and geographical location. Fifty percent of global candidemia cases were reported in Asia, followed by the Americas, Europe, and Africa (Kaur and Chakrabarti, 2017). In recent decades, the epidemiology has been changing, with a worrying increase in non-*C. albicans* species causing candidemia (Vaezi et al., 2017; Lamoth et al., 2018). However, in Latin America, *C. albicans* is still the most commonly found species causing this type of disease (da Matta et al., 2017). Infections of *C. albicans* have therefore become a serious public health problem with high mortality rates (Kett et al., 2011; Colombo et al., 2014; da Matta et al., 2017).

In general, the treatment of invasive fungal infections is restricted to three classes of major antifungal agents: azoles, polyenes and echinocandins. However, these drugs present disadvantages such as toxicity, the emergence of resistance, complex drug interactions, and significant limitations in activity (Pfaller, 2012; Vandeputte et al., 2012; Sanguinetti et al., 2015; da Matta et al., 2017). Due to this worrying scenario, the development of more specific antifungal agents is therefore necessary.

Rational drug design has allowed the application of *in silico* methodologies for drug discovery as a cost-effective alternative (Abadio et al., 2011, 2015; Salci et al., 2017). For these approaches, a promising drug target is usually an enzyme which is essential for the pathogen, however, it is absent in humans, conferring selective toxicity against infectious agent (Abadio et al., 2011).

Thioredoxin reductase (Trr1) is an important flavoenzyme, which participates in important processes for cellular maintenance, protecting cells against oxidative stress (Arneér and Holmgren, 2000; Holmgren, 2000). Two isoforms of Trr1 were characterized, but a low molecular weight isoform is only present in prokaryotes, plants, some parasites and fungi (Thön et al., 2007), thus confirming a selective target for the development of new drugs.

In previous work addressed by our research group, *in silico* methodologies allowed the selection of two new compounds that could be used against Trr1: 4-[benzyl(methyl)sulfamoyl]-N- [5-[ (4-methoxyphenyl ) methyl ]-1, 3, 4-oxadiazol-2-yl ] benzamide (LMM5) and 4-[cyclohexyl(ethyl)sulfamoyl]-N-[5-(furan-2-yl)-1,3,4-oxadiazol-2-yl]benzamide (LMM11) that belong to the class of 1,3,4-oxadiazoles which are denominated LMM5 and LMM11, respectively (Kioshima et al., 2018). Using assays for antifungal activity, we report that these two new 1,3,4-oxadiazoles class compounds, discovered by *in silico* methodologies, are possible thioredoxin reductase inhibitors and are effective against *C. albicans*.

## Materials and Methods

### Chemical Compounds

The 4-[benzyl(methyl)sulfamoyl]-N-[5-[(4-methoxyphenyl) methyl]-1,3,4-oxadiazol-2-yl]benzamide (LMM5) and 4-[cyclohexyl (ethyl) sulfamoyl] -N- [5- (furan-2-yl) -1 ,3 ,4-oxadiazol- 2-yl]benzamide (LMM11) compounds were purchased from Life Chemicals (F2368-0617 and F2832-0099) and solubilized in 0.5% dimethyl sulfoxide (DMSO) with the addition of 0.02% non-ionic surfactant Pluronic F-127 (P/F-127; Sigma). These diluents were included at the same concentrations as the control in all experiments. Fluconazole pure powder was acquired commercially from Pfizer.

### Fungal Strains and Growth Conditions

The antifungal activity of LMM5 and LMM11 compounds was evaluated against the reference strain *C. albicans* American Type Culture Collection (ATCC) 90028 and 15 clinical isolates from a mycology collection of the Medical Mycology Laboratory, Universidade Estadual de Maringá, Paraná, Brazil. Isolates were recovered from blood (*n* = 4), urine (*n* = 8) and catheters (*n* = 3). Other experiments were performed with the reference strain of *C. albicans* ATCC 90028. For each experiment, yeasts were previously subcultured on Sabouraud Dextrose Agar (SDA, Difco^tm^, Detroit, USAMI, United States) at 35°C for 24 h.

### Antimicrobial Susceptibility Testing

All isolates were tested against fluconazole, LMM5 and LMM11 compounds according to the Clinical and Laboratory Standards Institute protocol M27-A3 (Clinical, and Laboratory Standards Institute [CLSI], 2008), with modifications. The LMM5 and LMM11 concentrations ranged from 0.5 to 256 μg/ml and the final concentration of fluconazole ranged from 0.125 to 64 μg/ml. Fungal suspensions were measured spectrophotometrically at the 530 nm with 90 ± 2% transmittance. The final size of the stock inoculum was therefore achieved as 2-3 × 10^3^ colony-forming units (CFU)/ml, as determined by quantitative colony counts on SDA. The MIC results for all agents were evaluated after 24 h of incubation at 35°C. The Minimum Inhibitory Concentration (MIC) of compounds and fluconazole was considered as the lowest concentration with a 50% reduced cell growth in relation to the positive control, through visual and spectrophotometric readings. Three independent assays were performed. *C. krusei* (ATCC 6258) and *C. parapsilosis* (ATCC 22019) were used as the quality control isolates.

### Qualitative and Quantitative Analysis

For qualitative analysis of antifungal activity of LMM5 and LMM11, the minimum fungicidal concentration (MFC) was determined by inoculating each concentration from the MIC assay into SDA plates. The plates were then incubated at 35°C for 24 h. The MFC was defined as the lowest concentration of the compounds that significantly reduced yeast growth. In addition, for quantitative analysis, each compound concentration tested also determined the colony forming units per milliliter (CFU/ml).

### Assessment of LMM5 and LMM11 *in vitro* Cytotoxicity

The lineage cells Vero and HUVEC were cultivated at 37°C in a 5% CO_2_ and 95% air atmosphere. Dulbecco’s modified Eagle medium (DMEM; Gibco, MO, United States) was used for Vero, and for HUVEC complete RPMI 1640 with HEPES buffer (Roswell Park Memorial Institute, Gibco) was used. After >80% confluence, cells were trypsinized (Gibco) and the concentration was adjusted to 2 × 10^5^ cells/ml in each medium. Suspensions were then added to 96-well plates (TPP) and incubated overnight. After 24 h, wells were washed with phosphate-buffered saline (PBS), and exposed to different concentrations of LMM5 and LMM11 (0.5–256 μg/ml) in RPMI 1640 for 24 h. The control was only incubated with RPMI 1640 or DMEM. Cells were then washed with PBS and a cytotoxicity test was performed using the Cell Titer 96 assay (Promega, Madison, WI, United States), based on the reduction of MTS in DMEM without phenol red (Malich et al., 1997). Cytotoxicity of the compounds was evaluated as the mean of three independent experiments. The percentage of cell viability (%CV) was calculated using the following equation: %CV = (*A* sample/*A* blank) × 100, where blank is the medium with cells and MTS. The 50% cytotoxic concentration (CC_50_) was defined as the compound’s concentration (μg/ml) required for 50% reduction of cell viability.

### Antifungal Time–Kill Curve

*Candida albicans* ATCC 90028 (2-3 × 10^3^ yeast/ml) was grown in RPMI 1640 medium and was exposed to the following concentrations of LMM5 or LMM11: Sub-MIC (16 μg/ml), MIC (32 μg/ml) and 2xMIC (64 μg/ml). Two controls were prepared, one with only culture medium and the other with fluconazole (0.25 μg/ml). At predetermined time points (0, 2, 4, 6, 8, 12, 24 and 36 h), aliquots of 100 μl from each culture were withdrawn, diluted, plated onto SDA plates and incubated at 35°C for 24 h for CFU/ml determination. Fungistatic and fungicidal activities were defined as the reduction of <99.9% and ≥ 99.9% CFU/ml, respectively, when compared to the control value (Klepser et al., 1998).

### Ultrastructural Analysis

#### Scanning Electron Microscopy (SEM)

To observe morphological changes of *C. albicans*, caused by compounds, yeasts were grown (2-3 × 10^3^ yeast/ml) in RPMI medium and exposed to three LMM5 or LMM11 concentrations (Sub-MIC, MIC and 2xMIC) for 24 h at 35°C. Samples were adhered to the glass coverslips pre-coated with a thin layer of poly-L-lysine (Sigma Chemical Co., United States) and fixed with 2.5% glutaraldehyde in 0.1 M sodium cacodylate buffer, (4°C, pH 7.0). After 24 h, samples were dehydrated in an ethanol series (30, 50, 70, 90, and 100°GL), critical-point dried with CO_2_ (BALTEC CPD 030 Critical Point Dryer), coated with gold (BALTEC SDC 050 Sputter Coater), and observed under a scanning electron microscope (FEI Quanta 200, Netherlands) (de Oliveira et al., 2016).

#### Transmission Electron Microscopy (TEM)

*Candida albicans* ATCC 90028 (2-3 × 10^3^ yeast/ml) was treated with LMM5/LMM11 at MIC concentration for 24 h and then processed for transmission electron microscopy. Yeast cells were harvested, washed twice with PBS and fixed with 2.5% glutaraldehyde in 0.1 M sodium cacodylate buffer. Cells were then post-fixed in a solution containing 1% OsO_4_, 0.8% potassium ferrocyanide and 10 mM CaCl_2_ in 0.1 M cacodylate buffer, dehydrated in an increasing acetone gradient and embedded in Spurr resin (Low Viscosity Embedding Media Spurr’s Kit - #14300). The ultrathin sections were then stained with uranyl acetate and lead citrate, and images were obtained on a Zeiss 900 TEM.

#### Murine Model of Systemic Candidiasis

A mouse model of systemic candidiasis was established according to a previously described method by Wong et al. (2014), with modifications. For each compound 20 female BALB/c mice, at 6 weeks old, were divided into four groups (*n* = 5): treated with LMM5 (5 mg/kg), LMM11 (5 mg/kg), fluconazole (5 mg/kg) or diluent (PBS, DMSO and 0.02% F-127). The systemic candidiasis model of *C. albicans* was established by administering a 100 μl cell suspension of 5 × 10^5^ yeast cells (ATCC 90028) through the lateral tail vein. After 3 h of infection, the respective treatments were administered intraperitoneally according to the group, twice a day for 5 days. The mice were euthanized, and the kidney and spleen were aseptically removed, weighed and then homogenized in lysis buffer (200 mM NaCl, 5 mM EDTA, 10 mM Tris, 10% glycerol v/v, pH 8.30). The homogenates were serially diluted before plating on SDA plates and incubated at 35°C for 24 h. Fungal burden was expressed by the ratio of CFU/g of organs.

#### Histopathological Analyses

The kidney and spleen were fixed in 10% formalin and then processed, preserved in paraffin, and cut into 5-μm serial sections. The sections were stained by hematoxylin and eosin (H&E) and Gomori & Grocott and photographed using a binocular light microscope (Motic BA310- camera Moticam 5), at 400× and 600× magnification.

### Statistical Analysis

The data were analyzed using Prism 6.0 software (GraphPad, San Diego, CA, United States). *In vitro* cytotoxicity was analyzed using one-way analysis of variance (ANOVA) with the Bonferroni test. Fungal burden and quantitative analysis of MIC was analyzed using an unpaired *t*-test. All of the tests were performed with a 95% confidence level, and values of *p* < 0.05 were considered statistically significant.

## Results

### LMM5 and LMM11 Not Presented *in vitro* Toxicity in Two Different Cell Lines

**FIGURE 1:**
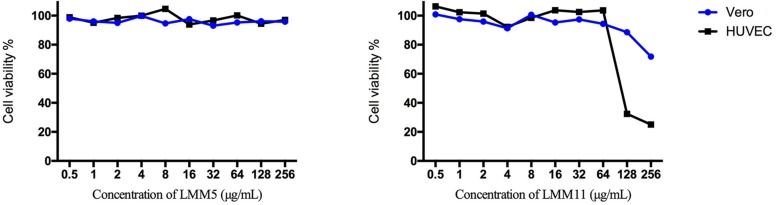
Cellular viability (%) of Vero and HUVEC cells in the presence of different concentrations of LMM5 and LMM11. The experiment was carried out with two cell lines by a colorimetric assay (MTS) at 24 h, evaluating concentration range 0.5 to 256 μg/ml. The 50% cytotoxic concentration (CC_50_) was defined as the compound’s concentration (μg/ml) required for the reduction of cell viability by 50%.

The *in vitro* cytotoxicity evaluation of LMM5 and LMM11 against two cell lineages is shown in Figure 1. No significant toxicity was observed for LMM5 at the concentrations analyzed. LMM11 reduced at least 50% cell viability (CC_50_) only in concentrations of 256 and 128 μg/ml for the HUVEC cell lineage. Considering that the average MIC values of these compounds are 32 μg/ml, the CC_50_ is 4- to 5-fold greater than the MIC. Diluent (DMSO + P/F-127) showed no significant toxicity in any of the evaluated parameters (data not shown).

### Both LMM5 and LMM11 Are Promising Antifungal Against *Candida albicans*

**FIGURE 2:**
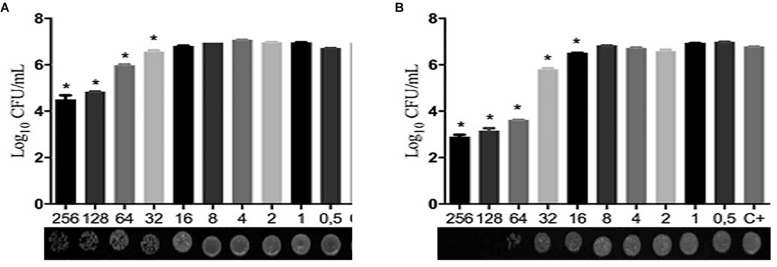
Quantitative and qualitative fungicide evaluation of LMM5 and LMM11 compounds against *Candida albicans.* Logarithm reduction of colony forming units (CFU) and minimum fungicidal concentration (MFC) after 24 h exposure to LMM5 **(A)** and LMM11 **(B)**. C + (Control): inoculum under the same conditions but without compounds, including diluents. ^∗^Values of *p* < 0.05 were considered statistically significant compared with control (C+).

Table 1 shows that the MIC values for two compounds were 32 μg/ml for the majority of *C. albicans* isolates tested. However, LMM11 exhibited a more homogeneous antifungal activity with 75% (12/16) of isolates having the same MIC value. Fluconazole presented MIC of 0.125 μg/ml (12/15) and 0.25 μg/ml (3/15) for clinical isolates, and 0.25 μg/ml for the reference strain. According to the qualitative analysis of *C. albicans*, LMM5 (Figure 2A) and LMM11 (Figure 2B) presented fungicidal activity with MFC values starting at 256 and 64 μg/ml, respectively. Moreover, through quantitative analysis, it was possible to observe a significant reduction (*p* < 0.05) of fungal growth in MIC concentrations, correlating with MIC values demonstrated in Table 1. For LMM11, this reduction was detected in a subinhibitory concentration (16 μg/ml).

**TABLE 1:** Inhibitory effect of LMM5 and LMM11 and standard antifungal fluconazole against several clinical isolates of *Candida albicans*.

***Candida albicans* (16)**	**MIC (μg/ml)**		
	
	**LMM5**	**LMM11**	**Fluconazole**
10	64	32	0.125
22	8	64	0.125
36	32	32	0.125
38	32	16	0.125
40	32	32	0.125
42	16	32	0.125
43	8	16	0.250
44	16	32	0.125
45	32	32	0.125
47	32	32	0.125
50	64	32	0.250
55	16	32	0.125
58	32	32	0.250
103	16	32	0.125
107	16	64	0.125
90028^a^	32	32	0.250

*Candida albicans: number of identifications for clinical isolates. ^*a*^*C. albicans* reference strain ATCC 90028. MIC, Minimum Inhibitory Concentration (Mean values of MIC are expressed by μg/ml obtained from three independent experiments).*

### LMM5 and LMM11 Are Fungistatic Against *Candida albicans*

**FIGURE 3:**
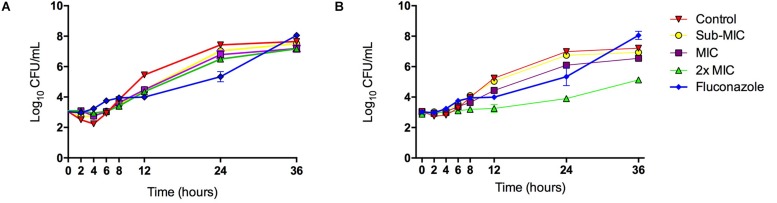
Killing kinetics of the LMM5 and LMM11 against *C. albicans*. Time-Kill curve of compounds: **(A)** LMM5 and **(B)** LMM11. The killing ability of the compounds was plotted from log_10_ CFU/ml versus time points. Standardized yeast cell suspensions were exposed to Sub-MIC (16 μg/ml), MIC (32 μg/ml) and 2xMIC (64 μg/ml) of LMM5 or LMM11. In time points 0, 2, 4, 6, 8, 12, 24, 28, and 36 h, aliquots were diluted and plated on SDA for CFU/ml determination. Absence of compounds was used as a positive control and the commercial antifungal fluconazole (0.25 μg/ml) was used for comparison. Data are representative of three independent experiments and each data point represents the mean ± standard deviation (error bars).

The inhibitory effect of LMM5 on *C. albicans* growth was observed 12 h after the start of incubation when compared to the control (Figure 3A). This effect remained for 24 h. For LMM11, according to Figure 3B, the inhibitory behavior also started at 12 h and remained for 36 h, but its effect was higher than observed for LMM5, especially at the 2xMIC concentration with a reduction of approximately two logs (log_10_) at 24 and 36 h. The best activity of commercial antifungal fluconazole was observed at 24 h in relation to the control, but the fungistatic effect was lost after 24 h. The compounds otherwise appear to maintain their effect until the last moment of observation. The fungistatic effect of LMM11 was better than observed to fluconazole independent of compound concentration at 36 h. Therefore, it was possible to observe that both compounds had better activity than fluconazole at time 36 h. *C. albicans* treated with LMM5 and LMM11 demonstrated endpoint activity (< 99.9% reduction in numbers of CFU/ml in relation to the control) at different time points, suggesting fungistatic activity like fluconazole.

### Ultrastructural Analysis Demonstrates Effective Activity of LMM5 and LMM11 in *Candida albicans*

**FIGURE 4:**
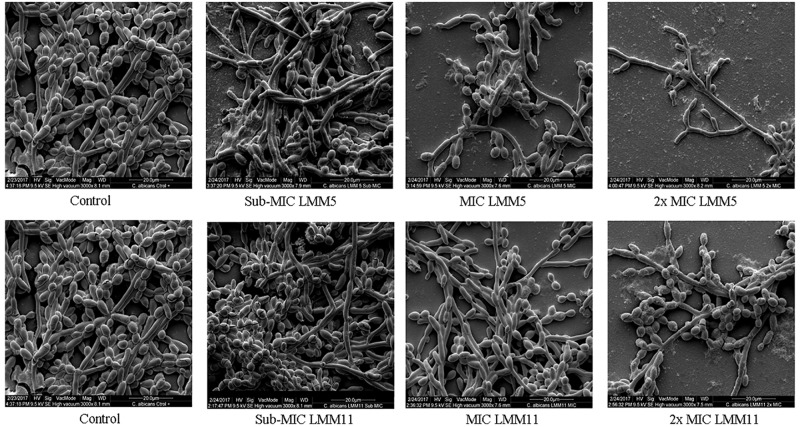
Scanning Electron Microscopy of *Candida albicans* reference strain after exposure to LMM5 and LMM11. Standardized yeast cells suspensions (2-3 × 10^3^ yeast/ml) were exposed to the LMM5 or LMM11 for 24 h/35°C in concentrations at Sub-MIC (16 μg/ml), MIC (32 μg/ml) and 2×MIC (64 μg/ml). Control: Absence of compounds. Magnification: 3000×.

**FIGURE 5:**
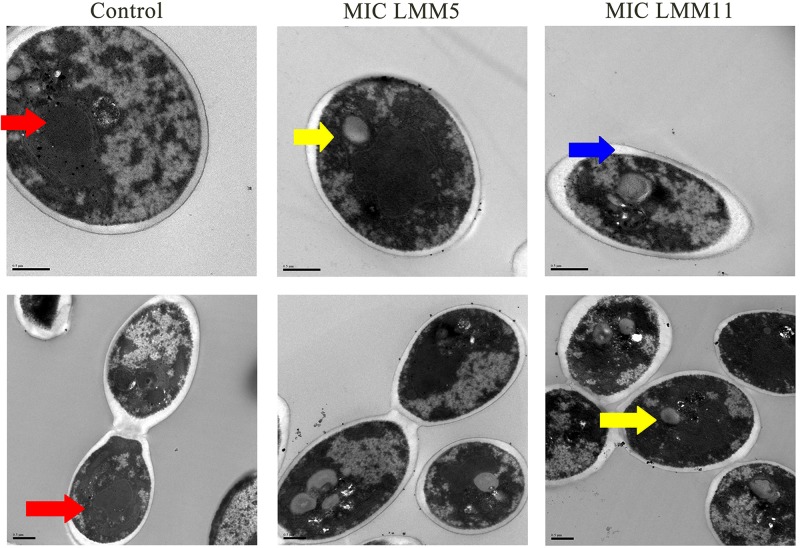
Transmission Electron Microscopy of *Candida albicans* reference strain after exposure to LMM5 and LMM11. Standardized yeast cells suspensions were treated with LMM5/LMM11 at 32 μg/ml (MIC) for 24 h and then processed for transmission electron microscopy. Images were obtained on a Zeiss 900 TEM. Control: Absence of compounds. Magnification: 15000× and 30000×. Red arrow: cytoplasm appeared homogeneous with a nucleus, mitochondria, surrounded by a defined cell membrane and regular cell wall; yellow arrow: large number of membranous bodies; blue arrow: alterations in the cell membrane and dysfunctions of the organelles.

Results of SEM showed that after 24 h in the presence of LMM5 or LMM11 at Sub-MIC, MIC and 2xMIC concentrations, the *C. albicans* population declined (Figure 4). External structural changes was not observed on *C. albicans*. Although proportional yeast growth decreased according to the increase of compounds concentrations, we suggest that the antifungal action of LMM5 and LMM11 is not related to mechanisms that cause structural morphological changes. Corroborating this observation, TEM photomicrographs (Figure 5) of the longitudinal and transverse sections of the untreated *C. albicans* control cells showed a homogeneous cytoplasm with a nucleus and mitochondria, surrounded by a defined cell membrane and regular cell wall. After 24 h of exposure to LMM5 and LMM11 in minimal inhibitory concentrations, it was possible to observe a large number of membranous bodies, notable alterations in the cell membrane, and dysfunctions of the organelles. A difference in electro density at the nucleus was also observed, suggesting changes in the genetic material. However, no change in cell wall was observed.

### LMM5 and LMM11 Decreased Fungal Burden in Murine Experimental Systemic Candidiasis

**FIGURE 6:**
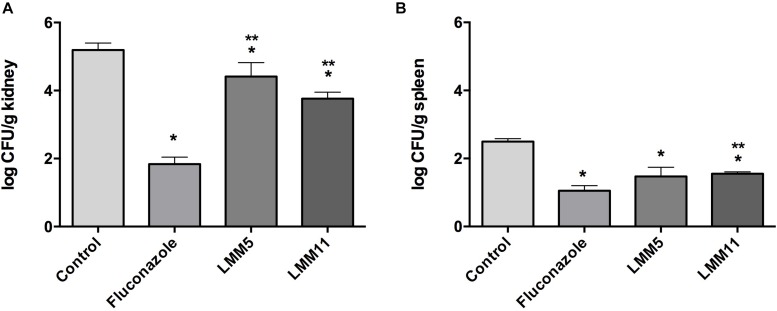
*In vivo* evaluation of antifungal activity of LMM5 and LMM11 compounds in murine systemic candidiasis by *Candida albicans.* For each compound 20 female BALB/c mice, at 6 weeks old were divided in four groups (*n* = 5) treated with: LMM5 (5 mg/kg), LMM11 (5 mg/kg), fluconazole (5 mg/kg) or diluent (PBS, DMSO, and 0.02% F-127). The systemic candidiasis model by *C. albicans* was established by administering 5 × 10^5^ yeast cells (ATCC 90028) through the lateral tail vein. After 3 h of infection, the respective treatments were administered intraperitoneally according to the group, twice a day for 5 days. **(A)** Colony Forming Units (Log_10_ CFU) per g of kidney. **(B)** Colony Forming Units (Log_10_ CFU) per g of spleen. The bars indicate the standard deviation. ^∗^Values of *p* ≤ 0.05 were considered statistically significant compared with control. ^∗∗^Values of *p* ≤ 0.05 were considered statistically significant compared with fluconazole.

**FIGURE 7:**
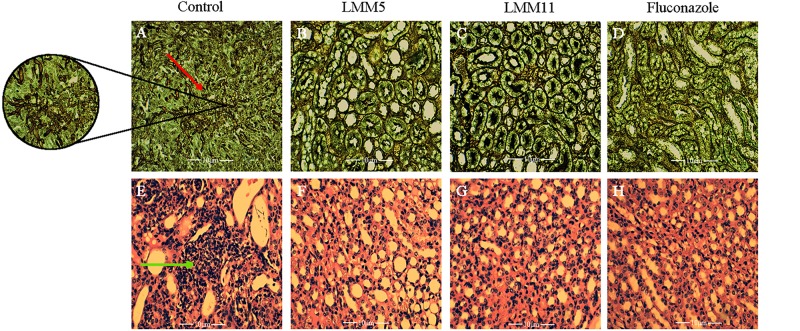
Histological sections of kidney from mice treated with LMM5, LMM11 and fluconazole and stained with Gomori & Grocott and hematoxylin and eosin (H&E). **(A–D)** Gomori & Grocott. **(E–H)** Hematoxylin and Eosin. **(A,E)** Histological sections of kidney treated with diluent (control group) and in larger magnification the agglomerate of yeasts (red arrow) and exacerbated inflammatory infiltrate in the respective region where yeasts were found in the control group (green arrow). **(B,F)** Histological sections of kidney treated with LMM5. **(C,G)** Histological sections of kidney treated with LMM11. **(D,H)** Histological sections of kidney treated with fluconazole. The histopathological samples were observed and photographed using a binocular light microscope (Motic BA310- camera Moticam 5), at ×400 and ×600 magnification.

The systemic candidiasis murine model of *C. albicans* was used for *in vivo* evaluation of antifungal activity. Treatment with LMM5 and LMM11 significantly reduced renal (Figure 6A) and spleen (Figure 6B) fungal burden in relation to the control (*p* < 0.05). Comparing the treatment groups, fluconazole versus new compounds, there was a significant difference in the kidney of the animals for both compounds, and only in the spleen for LMM11 (*p* < 0.05). Between the compounds, LMM11 had better inhibition on fungal burden in the kidney than LMM5 (0.5 log). Figure 7 demonstrates histopathological sections of the kidney that was treated with compounds and fluconazole. Analyzing histological sections stained with Gomori & Grocott, only the control group exhibited an abundant presence of yeasts, as shown in Figure 7A. In the animals from groups treated with LMM5 (Figure 7B), LMM11 (Figure 7C), and fluconazole (Figure 7D), rare or no yeasts were observed. In addition, the H&E staining indicated exacerbated inflammatory infiltrate in the equivalent region where yeasts were found in the control group (Figure 7E). On the other hand, no histological alterations were detected in the renal tissue of animals from other groups (Figures 7F–H). Some infiltration foci were also found in other groups, but in small numbers and extension (data not shown). As we can see in Figure 6, fungal burden in the spleen is less than that observed in the kidney, thus, the histology of the spleen was not demonstrated since no significant number of fungal cells was detected in the histological sections.

## Discussion

Considering the frequency of *C. albicans* as a pathogen responsible for systemic or invasive infections and the diversity of its virulence factors, the search for new compounds and targets addressed to this species is frequent. Regarding action in virulence factors, LaFleur et al. (2011) identified small molecules with activity against *C. albicans* biofilms and Toenjes et al. (2005) based on the ability to inhibit budded-to-hyphal-form transition. Targeting other mechanisms of action, Menzel et al. (2017) reported satisfactory *in vitro* and *in vivo* activity of a small molecule (C4) to treat invasive candidiasis. A study by Wong et al. (2014) characterized the novel antifungal small molecule (SM21) to treat local and systemic candidiasis.

In the search for specific targets, new compounds for candidiasis treatment has emerged in patent databases as inhibitors of Chitin synthase, Glucan synthase, Mannosyl transferase, and Secreted aspartyl proteases (SAPs) (Calugi et al., 2011). In this sense, Abadio et al. (2011) identified potential drug targets in human fungal pathogens. Among them, Trr1, which encodes an important flavoenzyme which participates in the redox state maintenance of the cell, and its low molecular weight isoform is present only in prokaryotes, plants, some parasites, and fungi (Thön et al., 2007), making it a selective target for the development of new drugs.

Using a set of *in vitro* and *in vivo* assays for antifungal activity, in this study, we report that LMM5 and LMM11, two inedit compounds of 1,3,4-oxadiazoles class (Kioshima et al., 2018), which were discovered by *in silico* methodologies as possible Trr1 inhibitors, were effective against *C. albicans*. The 1,3,4-oxadiazole is a heterocyclic compound containing an oxygen atom and two nitrogen atoms in a five-membered ring (de Oliveira et al., 2012). The compounds that contain this heterocyclic are widely studied by researchers because they have a broad spectrum of pharmacological activities including antifungal (Patel et al., 2013), antiviral (Lai et al., 2013) and antibacterial (Sekhar et al., 2018) activities.

Fungal cells present similarities with mammalian cells such as cytoplasmic organelles and biosynthetic pathways, making it difficult to target antifungal drugs that do not cause toxicity in humans (Krcmery and Kalavsky, 2007; Mazu et al., 2016). Interestingly, we demonstrate no significant *in vitro* toxicity for LMM5 at the concentrations evaluated and the CC_50_ values for LMM11 were at least 4- to 5-fold more than the MIC found (32 μg/ml). These results indicate that LMM5 and LMM11 can be used as antifungal agents without causing significant toxicity in human cells at concentrations that exceed the MIC. In addition, Rodrigues-Vendramini et al. (2019) demonstrated that both LMM5 and LMM11 showed no significant toxicity in *in vivo* assays with Balb/c mice and, both compounds have efficient activity against *Paracoccidioides* spp.

Fluconazole is a fungistatic antifungal drug commonly used in the treatment of *Candida* infections, presenting an effective spectrum of activity against these pathogens (Mushi et al., 2018). Recently, studies involving newly azole base compounds (Motahari et al., 2018; Mahmoudi et al., 2019) and combination therapy (Fakhim et al., 2017) demonstrate its antifungal activity against *C. albicans* and non *C. albicans* species. However, due to its frequent use, fluconazole resistant strains have emerged (Akins, 2005; Morschhäuser, 2016). A time-kill assay was employed to investigate the killing kinetics of both compounds compared to fluconazole. LMM5 and LMM11 also exhibited fungistatic activity against *C. albicans*. Nevertheless, at 36 h the compounds had a better effect than fluconazole (Figure 3).

In this view, it is interesting to think that the activity of LMM5 and LMM11 compounds, through the inhibition of Trr1, is more efficient in controlling fungal growth than the ergosterol biosynthesis pathway, which is performed by fluconazole.

The fungistatic profile of the compounds can also be observed through quantitative and qualitative analysis of MFC (Figure 1). Significant reductions in fungal growth were found with sub-MIC and MIC concentrations, for LMM11 and LMM5, respectively.

Still demonstrating the effective and similar activity of both compounds with fluconazole, LMM5 and LMM11 administered in the same dose than fluconazole (Louie et al., 1998), twice a day, beginning 3 h post-infection, were able to significantly reduce the renal and spleen fungal burden. In addition, the histological sections of the kidneys showed an abundant presence of yeasts only in the control group and an exacerbated inflammatory infiltrate in the same region. Models developed in mice with direct fungus inoculation through the tail vein, mimicking the systemic infection, are widely used and are considered “the Gold Standard” (Lopez-Berestein et al., 1984; Ikeda et al., 2000; Harwood and Rao, 2014; Salci et al., 2017). Wong et al. (2014) showed satisfactory effects of a new small molecule treatment in a systemic candidiasis mouse model. However, the authors concluded that it was complicated to compare effective doses in different studies because there was not a standard protocol and several parameters can be altered, such as inoculum, mouse immune status, the start and interval of antifungal therapy, and mouse strain. Besides that, it is very difficult to compare a developing compound with a commercially available drug, unlike fluconazole, which is already an established drug in terms of its formulation and pharmacokinetic and pharmacodynamic parameters, our two compounds are still considered hits in the pharmacological development stage.

In fact, in the next work on LMM5 and LMM11, pharmacokinetic and pharmacodynamic studies should be performed to establish more appropriate doses for *in vivo* treatment, relating drug time/concentration at sites of infection. Nevertheless, as shown in the time-kill curve, we can suggest a dose with an initial effect starting from 12 h.

Several studies have demonstrated the importance of Trr1 in pathogenic fungi during invasive diseases. Godoy et al. (2016) described the use of *C. albicans* Trr1 as a potential vaccine target and development of new drugs. In 2015, Abadio et al. (2015) performed molecular modeling of Trr1 from *P. lutzii*, and through the virtual library scanning of compounds, selected active compounds against flavoenzyme. Thioredoxin reductase has been shown to be essential for the viability of *C. neoformans* (Missall and Lodge, 2005). The *in silico* methodology enabled the discovery of Trr1 inhibitors such as, LMM5 and LMM11. This method has been widely used to search for new compounds through a known target for various diseases (Tripathi and Khan, 2018; Verma et al., 2018; Xia et al., 2018). According to the SEM and TEM images, exposure of *C. albicans* to compounds LMM5 and LMM11 were not related to structural morphological alterations, so we hypothesized that the mechanism of action for LMM5 and LMM11 is related directly to the inhibition of the enzyme Trr1 and it internally affects the fungal cell.

This is the first screening of *in vitro* and *in vivo* assays performed by these compounds against *C. albicans*. In general, both compounds had effective activity, however, LMM5 and LMM11, even belonging to the same chemical class (1,3,4 oxadiazoles) have structures that differentiate them, as presented in the study of Rodrigues-Vendramini et al. (2019), that can directly influence their activity in the established target. LMM11 showed more uniform MIC values, fungistatic action with lower concentrations, and greater inhibition of fungal burden in *in vivo* assays, which is more effective for the treatment of systemic candidiasis than LMM5. Despite this, considering that LMM5 also presented significant results and since these compounds are still hits, they can be extensively modified and improved upon until a marketable drug is obtained.

In view of the comprehensive set of *in vitro* and *in vivo* assays, our study revealed that LMM5 and LMM11 presented fungistatic activity against *C. albicans*, displayed low cytotoxicity, and decreased fungal burden in a murine model of experimental systemic candidiasis. These two compounds are therefore effective therapeutic candidates for the development of new antifungal drugs addressing the treatment of human infections caused by *C. albicans.*

## Data Availability

All datasets generated for this study are included in the manuscript and/or the supplementary files.

## Ethics Statement

All animal procedures were carried out in accordance with national regulations on animal experimentation adopted by the Brazilian Society of Laboratory Animal Science and they were approved by the Institutional Ethics Committee for Animal Experimentation of the Universidade Estadual de Maringá, Paraná, Brazil (Approval No. CEUA 9810191015, 04/22/2016).

## Author Contributions

IC, EK, and TS were involved in the study design presented in this manuscript. IC, DF, KS, FR-V, PB-M, EK, GA, and AO performed the experiments. IC, AO, EK, and TS analyzed the data. EK, TS, BM, and MF contributed reagents, materials, and analysis tools. The manuscript were produced by IC, EK, and TS with the assistance of all other co-authors listed.

## Conflict of Interest Statement

The authors declare that the research was conducted in the absence of any commercial or financial relationships that could be construed as a potential conflict of interest.
